# Subdoses of 17DD yellow fever vaccine elicit equivalent virological/immunological kinetics timeline

**DOI:** 10.1186/1471-2334-14-391

**Published:** 2014-07-15

**Authors:** Ana Carolina Campi-Azevedo, Paula de Almeida Estevam, Jordana Grazziela Coelho-dos-Reis, Vanessa Peruhype-Magalhães, Gabriela Villela-Rezende, Patrícia Flávia Quaresma, Maria de Lourdes Sousa Maia, Roberto Henrique Guedes Farias, Luiz Antonio Bastos Camacho, Marcos da Silva Freire, Ricardo Galler, Anna Maya Yoshida Yamamura, Luiz Fernando Carvalho Almeida, Sheila Maria Barbosa Lima, Rita Maria Ribeiro Nogueira, Gloria Regina Silva Sá, Darcy Akemi Hokama, Ricardo de Carvalho, Ricardo Aguiar Villanova Freire, Edson Pereira Filho, Maria da Luz Fernandes Leal, Akira Homma, Andréa Teixeira-Carvalho, Reinaldo Menezes Martins, Olindo Assis Martins-Filho

**Affiliations:** 1 Centro de Pesquisas René Rachou – Fiocruz, Belo Horizonte, Minas Gerais, Brazil; 2 Bio-Manguinhos, Fiocruz, Rio de Janeiro, Brazil; 3 Escola Nacional de Saúde Pública, Fiocruz, Rio de Janeiro, Brazil; 4 Instituto Oswaldo Cruz, Fiocruz, Rio de Janeiro, Brazil; 5 Instituto de Biologia do Exército, Rio de Janeiro, Brazil

**Keywords:** Yellow fever, Vaccine, Dose–response, Viremia, Cytokines, Ckemokines

## Abstract

**Background:**

The live attenuated 17DD Yellow Fever vaccine is one of the most successful prophylactic interventions for controlling disease expansion ever designed and utilized in larger scale. However, increase on worldwide vaccine demands and manufacturing restrictions urge for more detailed dose sparing studies. The establishment of complementary biomarkers in addition to PRNT and Viremia could support a secure decision-making regarding the use of 17DD YF vaccine subdoses. The present work aimed at comparing the serum chemokine and cytokine kinetics triggered by five subdoses of 17DD YF Vaccine.

**Methods:**

Neutralizing antibody titers, viremia, cytokines and chemokines were tested on blood samples obtained from eligible primary vaccinees.

**Results and discussion:**

The results demonstrated that a fifty-fold lower dose of 17DD-YF vaccine (587 IU) is able to trigger similar immunogenicity, as evidenced by significant titers of anti-YF PRNT. However, only subdoses as low as 3,013 IU elicit viremia kinetics with an early peak at five days after primary vaccination equivalent to the current dose (27,476 IU), while other subdoses show a distinct, lower in magnitude and later peak at day 6 post-vaccination. Although the subdose of 587 IU is able to trigger equivalent kinetics of IL-8/CXCL-8 and MCP-1/CCL-2, only the subdose of 3,013 IU is able to trigger similar kinetics of MIG/CXCL-9, pro-inflammatory (TNF, IFN-γ and IL-2) and modulatory cytokines (IL-5 and IL-10).

**Conclusions:**

The analysis of serum biomarkers IFN-γ and IL-10, in association to PRNT and viremia, support the recommendation of use of a ten-fold lower subdose (3,013 IU) of 17DD-YF vaccine.

## Background

The yellow fever (YF) virus is a mosquito-borne flavivirus that causes hemorrhagic disease with jaundice in people inhabiting tropical areas [1-3]. The incidence of YF dropped significantly after the development of live attenuated vaccines in the 1930s [3]. The YF vaccines (17D and 17DD) are one of the most successful prophylactic interventions for controlling disease expansion ever designed and utilized in larger scale [4].

Recent evidences for expansion of viral circulation in tropical areas estimate that over 900 million people are at risk of infection [5]. It is now effective that the YF vaccine is been currently used to protect travelers and is incorporated in childhood vaccination programs in many countries, with millions of doses distributed annually around the globe. Up to present, there are 6 producers of yellow fever vaccines, but only 4 are prequalified by the World Health Organization and supply vaccines to international agencies: Bio-Manguinhos (Brazil), Sanofi-Pasteur (France), Institute Pasteur in Dakar (Senegal) and Chumakov Institute - Institute of Poliomyelitis and viral encephalitides (Russia Federation) [6]. In 2008, the sudden increase on the demand for YF vaccine forced Bio-Manguinhos, the Brazilian supplier of 17DD vaccine, to interrupt any vaccine exporting to other countries [7]. This sudden rise in vaccine demand was due to the large epidemic of yellow fever in tropical South America, caused by the increase of *Aedes aegypti* infestation levels in many urban cities, in addition to the frequent movement of susceptible individuals from yellow fever-free to endemic areas [7].

Thus, the spreading of risk areas and the restricted group of YF vaccine manufacturers creates a shortage on YF vaccine supply worldwide, which urges for new strategies of vaccination protocols including validation of new seed lots, need and timing of booster doses to maintain long lasting protection as well as dose sparing studies [8]. In regards to dose, the minimal number of viral particles has been established by WHO as at least 5,000PFU or approximately 3,000 IU. However, the maximum dose has not been established [5,9]. Previous studies have reported that the number of virions in the 17DD-YF vaccine produced by Bio-Manguinhos/FIOCRUZ is on average approximately seven times higher (2.3 to 12.0 times) than the minimal dose established by WHO [5,9]. The fine-tuning of the vaccine dose in current use to lower number of viral particles, above the minimum required by WHO, could increase the vaccine availability and supply the worldwide increasing needs. However, it is important to guarantee that lower doses are able to induce similar protection [9]. It has been proposed by Lopes et al. [10] that doses higher than 200 PFU (approximately 100 IU) were able to induce 100% of seroconversion. However, recent evidence has shown that doses as low as 47 times (1,122PFU or 587 IU) the reference are required to induce equivalent seroconversion rates [5,9]. It is clear that a better understanding of the virological/immunological features upon YF subdoses vaccination is relevant to further support changes in the minimal dose recommended by the YF-vaccination guidelines. Therefore, in the present study, individuals who had primary vaccination with subdoses of the 17DD-YF vaccine were tested for virological/immunological serum biomarkers, such as the viral load, chemokines and cytokines as well as neutralizing antibody titers. The kinectics of such biomarkers, taken in association, highly advice for alternative and equivalent vaccination protocols with subdoses of the 17DD-YF vaccine.

## Methods

### Design of the study

**Figure 1:**
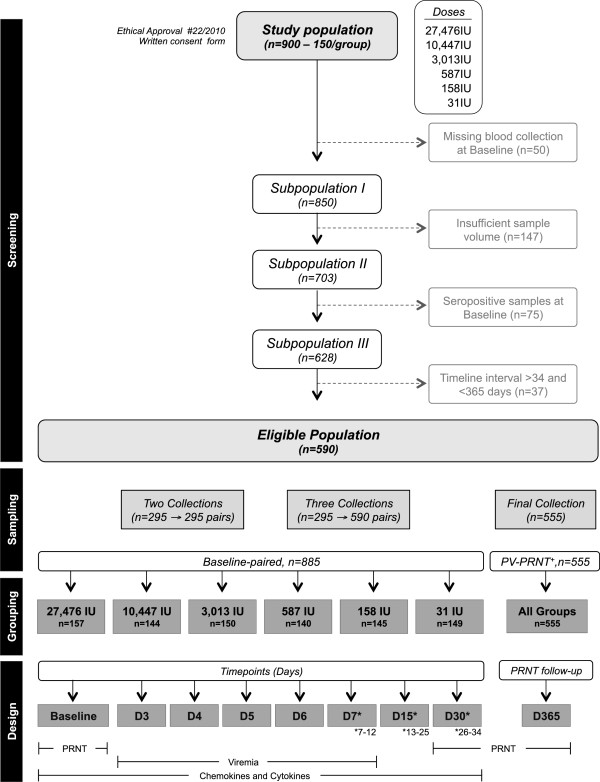
**Flowchart illustrating the study population and experimental design.** The study was organized in four phases: Screening, Sampling, Grouping and Design. “Screening” of volunteers was performed taking into account: i) missing blood collection at baseline (n = 50); ii) insufficient sample volume (n = 147); iii) seropositivity at baseline (n = 75) and iv) timeline interval of blood collection >34 days and <365 days after primary vaccination (n = 37). The eligible population comprises 590 primovaccinees. “Sampling” consisted of either two (n = 295) or three (n = 295) blood collections. The serum samples were paired to their respective sample collected at baseline, resulting in a total of 885 baseline-paired samples. “Grouping” was performed according to the dose of 17DD YF vaccine administered (27,476 IU; 10,447 IU; 3,013 IU; 587 IU; 158 IU and 31 IU). Paired samples were further subcategorized according and the timepoint (days) in which the sample was collected (Baseline; D3; D4; D5; D5; D7(7–12); D15(13–25) and D30(26–34). A final blood collection of these volunteers was taken at 365 days after primary vaccination (n = 555) to monitor anti-YF antibody status. “Design” included: i) PRNT assays performed at baseline, D30 and D365; ii) Viremia quantified at D3, D4, D5, D6 and D7 and iii) Kinetics of serum chemokines and cytokines evaluated from baseline to D30.

*The* present study was performed by the Collaborative Group for Studies of Yellow Fever Vaccine aiming to investigate virological and immunological features induced by subdoses of the 17DD-YF Vaccine after approval of the Ethical Committee for studies with human subjects (CPqRR/FIOCRUZ #22/2010). The study population consisted of 900 healthy, adult (age average - 19.4 years), army, male conscripts from Rio de Janeiro enrolled in a screening phase. All participants informed not being vaccinated for Yellow Fever previously and agreed with and signed a written consent form. Participants were distributed randomly into six study groups (150 subject/group) each of which were given different the currently used dose of 17DD-YF vaccine (27,476 IU - 52,480PFU) and five alternative formulation with decreasing number of viral particles (10,447 IU - 19,953PFU; 3,013 IU - 5,754PFU; 587 IU - 1,122PFU; 158 IU - 302PFU and 31 IU - 59PFU), as shown in Figure 1. Excluding criteria were: 1) missing blood collection at Baseline (n = 50), 2) insufficient serum sample volume (n = 147), 3) Seropositivity (PRNT ≥ 2.70 log_10_ mIU/mL) at Baseline (n = 75) or 4) timeline interval of blood collection >34 days (n = 37). The eligible population (n = 590) was selected for pairing with baseline sample according to the number of blood samples available (two blood samples, n = 295 → 295 pairs and three blood samples, n = 295 → 590 pairs), resulting in a total of 885 paired samples. Paired samples were grouped according to dose given and referred as 27,476 IU (n = 157), 10,447 IU (n = 144), 3,013 IU (n = 150), 587 IU (n = 140), 158 IU (n = 145) and 31 IU (n = 149). The experimental design consisted of eight timepoints: before (Baseline) and days after primary vaccination (D3, D4, D5, D6, D7, D15 and D30). Each timepoint was comprised in average of 21 paired samples for each dose. The Plaque Reduction Neutralization Test (PRNT) was performed at baseline and D30. Viremia was assayed at D3, D4, D5, D6 and D7. Chemokines and cytokines were evaluated at Baseline, D3, D4, D5, D6, D7, D15 and D30. The volunteers who showed negative PRNT results at D30 (n = 35) were revaccinated with the current vaccine. PRNT follow-up of those volunteers with positive PRNT results after primary vaccination (PV-PRNT^+^, n = 555) was performed in a final blood sample collected at endpoint (D365).

### Neutralizing antibody test – PRNT

The antibody titer against Yellow Fever virus was defined by the Plaque Reduction Neutralization Test (PRNT) performed at Virological Technology Laboratory of Bio-Manguinhos (LATEV, FIOCRUZ). This test was conducted as previously described [9,11]. Briefly, sera samples from the participants were separated, inactivated and prepared as 2-fold dilution starting at 1:5, in volumes of 50 μl of samples in flat-bottom 96-well tissue culture plates. Twenty-five plaque-forming units (PFU) of yellow fever virus (strain 17D 213/77, lot UEXVFB01, Dec 1011) in 50 μl were added to all wells. A positive serum sample with anti-Yellow Fever virus antibody, properly calibrated by a WHO International Reference Preparation agency, was included in each set of the test. Using linear regression, the log_10_ dilution of the test and standard serum that reduced the plaque numbers in 50% relative to the virus control was determined. The mean antibody titer at the 50% end-point of the standard serum is then calculated and added to the log_10_ end-point for each sample in order to obtain the values in log_10_ mIU/ml. Results are presented in log_10_ mIU/mL. The 2.7 log_10_ mIU/mL cut-off point was applied to segregate seropositive from seronegative samples as described previously [9].

### Viremia

The RNA of Human serum samples from vaccinated patients with Yellow Fever vaccine was extracted using QIAamp® Viral RNA Mini kit (QIAGEN®) according to the manufacturer’s recommendations. Reverse transcription reaction was performed with random primers (F: 5′GCACGGATGTAACAGACTGAAGA3′ and R: 5′CCAGGCCGAACCTGTCAT 3′) in 20 μL of the extracted RNA added to 20 μL of High-Capacity cDNA Reverse Transcription mix (Applied Biosystems®). The qRT-PCR assays were performed in the ABIPrism 7500 (Applied Biosystems®) using the probe Fam-CGACTGTGTGGTCCGGCCCATC-Tamra, directed to the NS5 region of the yellow fever virus [12,13]. For constructing the standard curves, the 83 bp fragments from the NS5 viral region obtained by PCR [12] was cloned into a TOPO TA Cloning vector, according to the manufacturer’s instructions (Invitrogen®). Serial dilutions from 10^7^ to 10^2^ plasmid copies per reaction were used to generate the calibration curves for the qRT-PCR assays. The lowest detected concentration was established in 25 copies/reaction.

### Chemokine and cytokine quantitation by flow cytometry - CBA

To assess the levels of chemokines – IL-8/CXCL-8, MCP-1/CCL-2, MIG/CXCL-9, IP-10/CXCL-10 and cytokines – TNF, INF-γ, IL-2, IL-4, IL-5 and IL-10 – in serum samples from 17DD-YF vaccinees, Cytometric Bead Array kits (BD Biosciences, California, USA) were used according to manufacturer’s protocol and adapted as described previously [14]. Analysis of raw data was performed using the FlowJo cytometry analysis software (FlowJo, Stanford, USA) and the mean fluorescence intensity (MFI) of each bead cluster was evaluated to calculate the each cytokine concentration in the sera of patients. Cytokines concentrations were extrapolated according to the standard curve created by serial dilutions of the positive control. The final data for the kinetics timeline design were expressed as fold changes based on the baseline concentration for all chemokines and cytokines tested.

### Data analysis

The overall kinetics profile of viremia, chemokine and cytokines elicited by 17DD-YF vaccine subdoses were evaluated in comparison to the current dose (27,476 IU) as standard. Equivalence between current dose and subdoses was considered when the median fold change at a given timepoint did not differ as compared to the current dose (Man-Whitney test). The peaks of fold changes along the timeline were also taken into account as a relevant feature for equivalence assessment and highlighted by *. The analyses of PRNT titers were performed in a categorical fashion using the cut-off point of 2.7 log_10_IU/mL as a threshold to segregate positive from negative results. Gray-colored rectangles were used to highlight equivalence in the kinetics profiles. The Prism GraphPad Software version 5.0 (San Diego, CA, USA) and Microsoft Office Excel 2010 were used for kinetics timeline graphic arts and data mining.

## Results

### A fifty-fold lower dose of 17DD-YF vaccine is able to trigger similar immunogenicity as evidenced by significant titers of anti-yellow fever virus neutralizing antibodies

The current dose of Yellow Fever vaccine distributed by Bio-Manguinhos is 27,476 IU, which is approximately seven times higher than the dose recommended by WHO. Therefore, in order to investigate the immunogenicity of lower doses of the 17DD Yellow Fever vaccine, a dose-sparing study was designed and performed including six doses of 17DD-YF vaccine as shown in Figure 1.

**Figure 2:**
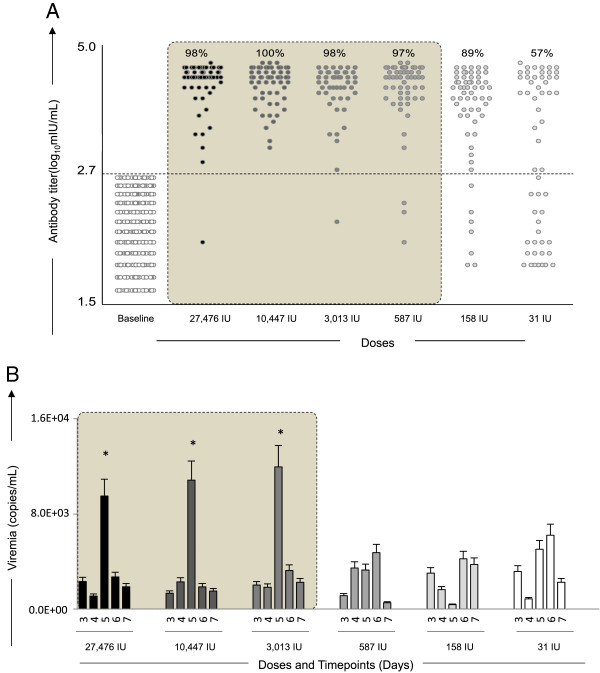
**Immunogenicity and Viremia kinetics following 17DD-YF primary vaccination with different doses (27,476 IU-current; 10,447 IU; 3,013 IU; 587 IU; 158 IU and 31 IU). (A)** Anti-YF neutralizing antibody titers (• = current dose and fades for subdoses) were measured by PRNT assay carried out 26–34 days (D30) after primary vaccination, as described in Methods. PRNT antibody titers are expressed in log_10_ mIU/mL and 2.7 log_10_ mIU/mL as the cut-off point to segregate seropositive from seronegative samples. Significant seropositivy rates (>95%) are highlighted by gray rectangle. **(B)** Viremia ( = current dose and fades for subdoses) was quantified by Real-time PCR at D3, D4, D5, D6 and D7 after primary vaccination as described in Methods. Viremia results are expressed in copies/mL. Kinetics profiles equivalent to current dose (27,476 IU) are highlighted by gray rectangle. The peaks of fold changes along the timeline were also taken into account as a relevant feature for equivalence assessment and highlighted by *.

Figure 2A shows the results on the Plaque Reduction Neutralization Test (PRNT) in sera samples of volunteers vaccinated by several doses of 17DD Yellow Fever vaccine. The threshold of 2.70 log_10_ mIU/mL was used as a cut-off to segregate seropositive from seronegative samples. The results show that seropositivity was high among vaccinees of all doses with percentages above 97% of seroconversion down to dose 587 IU of YF vaccine. At doses 158 IU and 31 IU, the percentage of seroconversion was 89% and 57%, respectively.

Additional analysis of PRNT at D365 indicated that only the lower dose (31 IU) was associated with significant seroreversion. In fact, only 89% of primary vaccinees that received the 31 IU subdose persisted with positive PRNT results one year after primary vaccination (Additional file 1: Figure S1).

### A ten-fold lower dose of 17DD-YF vaccine elicits equivalent viremia kinetics with an early peak at five days after primary vaccination

Figure 2B shows the kinetics timeline of Viremia following 17DD-YF primary vaccination with the dose currently used and subdoses. It is possible to observe a significant viremia peak at D5 after primary vaccination with the doses 27,476 IU, 10,447 IU and 3,013 IU. The kinectics of viremia at the lower doses is distinct, with a late and lower-magnitude peak at D6 post-vaccination.

**Figure 3:**
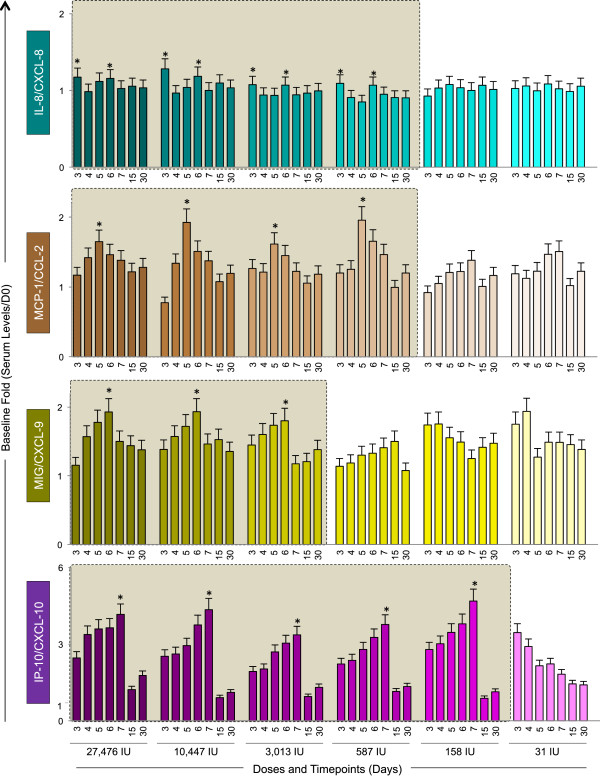
**Kinetics of serum chemokines following 17DD-YF primary vaccination with different doses (27,476 IU-current; 10,447 IU; 3,013 IU; 587 IU; 158 IU and 31 IU).** Serum levels of IL-8/CXCL-8 (), MCP-1/CCL-2 (), MIG/CXCL-9 () and IP-10/CXCL-10 () were measured by cytometric beads array (CBA) as described in Methods. The colors assigned for the current dose fade away for subdoses. Results are expressed as baseline fold change of each timepoint (Serum level/D0). Kinetics profiles equivalent to current dose (27,476 IU) are highlighted by gray rectangle. The peaks of fold changes along the timeline were also taken into account as a relevant feature for equivalence assessment and highlighted by *.

A Fifty-fold lower dose of 17DD YF vaccine is able to trigger equivalent kinetics of IL-8/CXCL-8 and MCP-1/CCL-2 as observed for PRNT seropositivity.Figure 3 shows the results of IL-8/CXCL-8 and MCP-1/CCL-2 kinetics in sera samples from Yellow Fever vaccinees. The chemokine results are displayed as Baseline fold changes for each timepoint. Data analysis demonstrates that doses as low as 587 IU are able to induce similar kinetics for IL-8/CXCL-8 and MCP-1/CCL-2, characterized by peaks at D3 or D6, and D5 respectively.

### The MIG/CXCL-9 kinetics induced by a ten-fold downscaled dose resembles the current dose as observed for the viremia kinetics

Figure 3 also shows the results of MIG/CCL-9 and IP-10/CXCL-10 kinetics in sera samples from Yellow Fever vaccinees. For the MIG/CCL-9, similar increasing kinetics with peak at D6 was observed upon vaccination with doses as low as 3,013 IU specifically one day after the viremia peak (Figures 2 and 3, respectively). The chemokine IP-10/CXCL-10 displayed the highest baseline fold change magnitude (up to 6 times) with peak of production at D7, except at the lowest dose of vaccine (31 IU), which showed an inverted kinetics profile.

**Figure 4:**
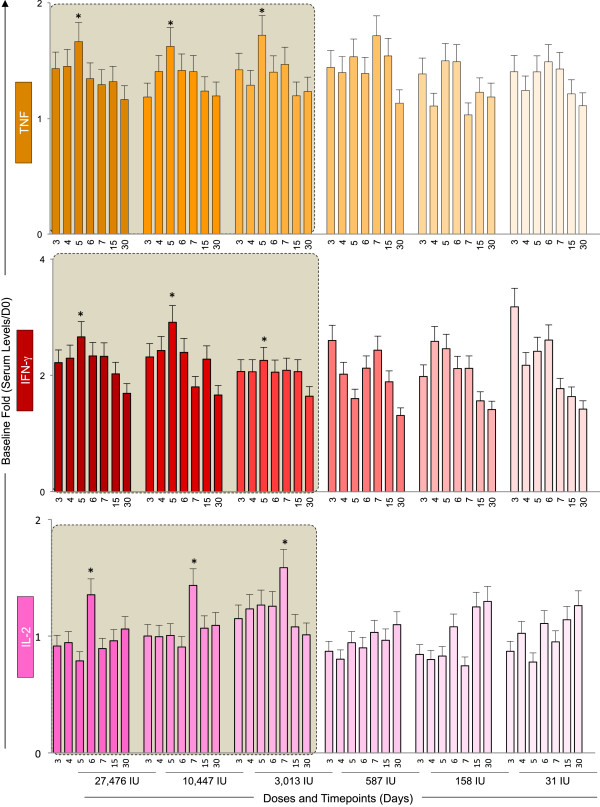
**Kinetics of pro-inflammatory cytokines following 17DD-YF primary vaccination with different doses (27,476 IU-current; 10,447 IU; 3,013 IU; 587 IU; 158 IU and 31 IU).** Serum levels of the pro-inflammatory cytokines TNF (), IFN-γ () and IL-2 () were measured by cytometric beads array (CBA) as described in Methods. The colors assigned for the current dose fade away for subdoses. Results are expressed as baseline fold change of each timepoint (Serum level/D0). Kinetics profiles equivalent to current dose (27,476 IU) are highlighted by gray rectangle. The peaks of fold changes along the timeline were also taken into account as a relevant feature for equivalence assessment and highlighted by *.

The kinetics of pro-inflammatory cytokines (TNF, IFN-γ and IL-2) progresses similarly down to a ten-fold lower dose of 17DD-YF vaccine.Figure 4 displays the results of the baseline fold changes in the serum pro-inflammatory cytokines TNF, IFN-γ and IL-2. The kinetics of these cytokines demonstrates similar peak of TNF and IFN-γ at D5 after vaccination with the doses 27, 476IU, 10,447IU and 3,013IU in agreement with the peak of viremia. IL-2 production display peak approximately one day after TNF and IFN-γ (D6 or D7) for the these three higher doses. Conversely, the IL-2 kinetics induced by the three lowest doses (587IU, 158IU and 31IU) is characterized by a shift of IL-2 peak towards D30.

### Peaks and troughs of IL-10 serum levels upon vaccination with 17DD YF vaccine – a distinguished modulatory cytokine kinectics

**Figure 5:**
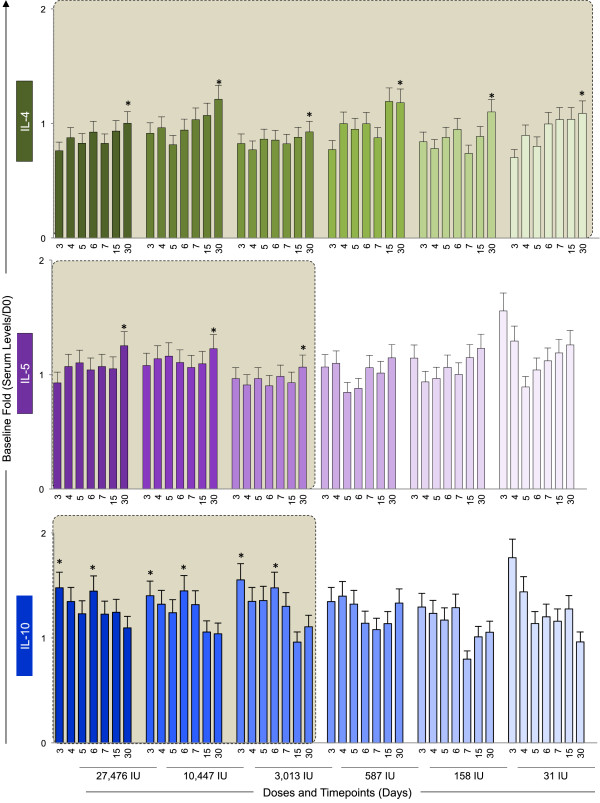
**Kinetics of regulatory cytokines following 17DD-YF primary vaccination with different doses (27,476 IU-current; 10,447 IU; 3,013 IU; 587 IU; 158 IU and 31 IU).** Serum levels of the regulatory cytokines IL-4 (), IL-5 () and IL-10 () were measured by cytometric beads array (CBA) as described in Methods. The colors assigned for the current dose fade away for subdoses. Results are expressed as baseline fold change of each timepoint (Serum level/D0). Kinetics profiles equivalent to current dose (27,476 IU) are highlighted by gray rectangle. The peaks of fold changes along the timeline were also taken into account as a relevant feature for equivalence assessment and highlighted by *.

Figure 5 demonstrates the results of baseline fold changes for the regulatory cytokines (IL-4, IL-5 and IL-10). The IL-4 secretion pattern was very similar throughout the subdoses, with late peak at Day 30 regardless of the vaccine dose given. For IL-5 and IL-10, similar kinetics profiles were observed to doses as low as 3,013 IU. However, IL-10 production presented a distinguished modulatory cytokine kinectics with early peak at day 3 with subsequent trough after day 4 or day 5 that upsurges at day 6, whereas IL-5 peaks at day 30 as observed for IL-4. Lower doses of the vaccine do not present the specific IL-10 peak and through kinetics, but rather present inverted pattern with increased IL-10 production in either earlier or later timepoints.

**Figure 6:**
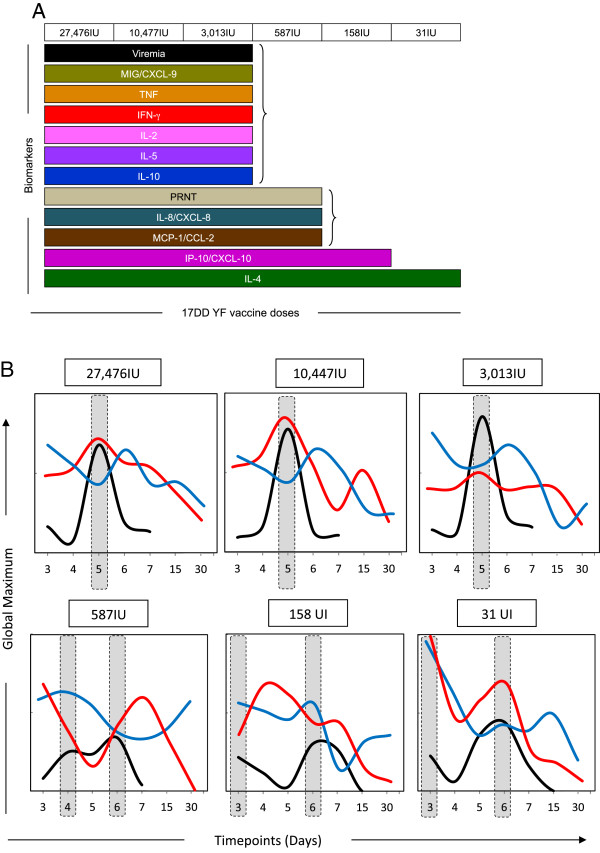
**Snapshot of immunological and virological biomarkers following 17DD-YF primary vaccination with different doses (27,476 IU-current; 10,447 IU; 3,013 IU; 587 IU; 158 IU and 31 IU). (A)** Selection of biomarkers with equivalent kinetics timeline with viremia and PRNT [Viremia (); PRNT (); IL-8/CXCL-8 (); MCP-1/CCL-2 (); MIG/CXCL-9 (); IP-10/CXCL-10 (); TNF (), IFN-γ () and IL-2 ();IL-4 (), IL-5 () and IL-10 ()]. **(B)** Overlay graphs highlighting that IFN-γ and IL-10 are relevant complementary biomarkers associated with the viremia kinetics. Results are expressed as the global maximum values of each timepoint to baseline (viremia, , range 0 to 1.6E + 04 copies/mL; INF-γ, , range 0 to 4 baseline fold; IL-10, , range 0 to 2 baseline fold).

IFN-γ and IL-10 are relevant complementary biomarkers that in addition to PRNT and viremia support a safe use of ten-fold lower subdose of 17DD YF vaccineThe overall analysis of serum chemokine and cytokines in addition to PRNT and viremia allowed the identification that six out of ten biomarkers (MIG/CXCL-9, TNF, IFN-γ, IL-2, IL-5 and IL-10) display equivalent kinetics timeline with viremia and PRNT (Figure 6A). On the other hand, biomarkers (IL-8/CXCL-8 and MCP-1/CCL-2) were demonstrated as similar in kinetics with the PRNT positivity. Two biomarkers (IP-10/CXCL-10 and IL-4) did not display any similarity with PRNT and viremia, the current conventional correlates of protection (Figure 6A).It is important to highlight that IFN-γ and IL-10 are relevant complementary biomarkers associated with the viremia kinetics. Together, these biomarkers in addition to PRNT and viremia could support the safe use of ten-fold lower subdose (3,013 IU) of 17DD YF vaccine (Figure 6B). The 3,013 IU subdose induced IL-10 trough simultaneously to viremia and IFN-γ peaks at D5, similar to the dose currently used (Figure 6B).

## Discussion

In the present study, individuals vaccinated with subdoses of 17DD vaccine were tested for several molecules such as cytokines, chemokines as well as the viral load and the neutralizing antibody titers in a time and dose-dependent fashion. The kinectics of these molecules were thoroughly investigated taking into consideration several vaccine doses and immune biomarkers of effective vaccination were proposed.

The results demonstrated that a fifty-fold lower dose of 17DD-YF vaccine (587 IU) is able to trigger similar immunogenicity as evidenced by significant titers of anti-YF PRNT. However, it is important to consider whether the load of antigen exposure during primary vaccination would affect immunogenicity. Only subdoses as low as 3,013 IU elicit viremia kinetics with an early peak at five days after primary vaccination equivalent to the current dose (27,476 IU), while other subdoses show a distinct, lower in magnitude and later peak at day 6 post-vaccination. It is well described that an optimal antigen exposure is necessary for the generation of protective response. In the context of live-attenuated virus, lower doses of vaccine should be able to elicit ideal immunogenicity since the virus replicates within the organism and amplifies its antigenic exposure. This fact indicates that viremia results should be taken together with PRNT titers on the decision making for dose sparing [9,10,15].

In addition to that, although subdoses as low as 587 IU is able to trigger equivalent kinetics of IL-8/CXCL-8 and MCP-1/CCL-2, only subdoses downscaled to 3,013 IU are able to trigger similar kinetics of MIG/CXCL-9, pro-inflammatory (TNF, IFN-γ and IL-2) and modulatory cytokines (IL-5 and IL-10).

It is important to note that our results indicate that even subdoses of 17DD yellow fever vaccine have the proper amount of YF vaccine virus to compensate for the possible loss during transportation of the vaccine or when the cold chain is not secured. In addition, the vaccine formulation is comprised of sorbitol and gelatin, which are used as vaccine stabilizers, and the vaccine preparation is lyophilized, allowing for more stability of the vaccine formulation.

Previous studies have demonstrated that the balance of cytokines is crucial to generate a pro-inflammatory response upon vaccination [16-22]. In this regard, TNF-α is an important molecule in this scenario. TNF-α produced by neutrophils and monocytes upon antigenic stimuli seem to play a role in inducing an initial pro-inflammatory response in either primo-vaccination or upon re-vaccination [22]. The levels of this cytokine also correlate with the PRNT titers, which indicates that the production of TNF-α may be important to generate an immunological environment for production of neutralizing antibodies [22].

Conversely, the lack of seroconversion after YF-17DD primary vaccination promotes a regulatory status upon antigen recall, with lower synthesis of TNF-α by neutrophils and monocytes. In addition, the absent production of neutralizing antibodies correlates with enhanced synthesis of modulatory cytokines such as IL-10 produced by CD8^+^ T cells compared with all other groups [22].

These results are in agreement with the present findings that indicate a peaks and troughs in IL-10 levels that are opposite to viremia peaks and peak levels of TNF-α and IFN-γ. The trough of IL-10 levels at day 5 may be associated with retreated modulatory response during peak viremia, which allows for the development and maturation of antigen presenting cells (APCs) as well as higher expression of MHC in the surface of these APCs [19]. The assembly of neutralizing antibodies is dependent upon antigenic presentation, mediated by MHC-II presentation by APCs, which endorses the data associating lower IL-10 synthesis, and higher TNF-α and IFN-γ production with PRNT titers and Viral load (Figure 6). The late IL-2 production may be associated with proliferation and clonal expansion of memory CD4^+^ and CD8^+^ T cells [21].

The kinetics observed in this study was very similar to the one found in previous study demonstrating early and strong cytokine production on 5–7 days after vaccination [18]. The overall cytokine secretion kinetics of 17DD YF vaccinees showed a transient peak of pro-inflammatory molecules and viral load along with trough of IL-10 secretion at day 5, which draws back toward a mixed/regulatory pattern at later times points in day 15 and day 30. A robust early response is important for maintaining a protective response as indicated by neutralizing antibodies.

Regarding the standardization of the serum biomarkers, we believe that the measuring of viremia, cytokines and chemokines could be easily standardized for regular use. Our group has advanced in this respect as previously described [14]. In addition, Chao et al. [13] and Jonsson et al. [23] have already described the correlation between the quantitation of YFV by real-time PCR and TCID50 or plaque assay lysis, which indicates that viral load could be implemented as a test with high likelihood to plaque assay lysis [13,23].

All in all, the results indicate that subdoses of 17DD vaccines are able to elicit neutralizing antibodies, peak viremia and strong pro-inflammatory response in a timeline similar to the one observed with a higher dose in current used.

## Conclusions

The analysis of serum biomarkers IFN-γ and IL-10, in association to PRNT and viremia, support the recommendation of use of a ten-fold lower subdose (3,013 IU) of 17DD-YF vaccine.

## Abbreviations

APCs: Antigen presenting cells; CCL-9: Chemokine receptor type nine; CCL-2: Chemokine receptor type two; CD4: Cluster of differentiation four; CD8: Cluster of differentiation eight; CNPq: Conselho Nacional de Desenvolvimento Científico e Tecnológico; CPqRR: Centro de Pesquisas René Rachou; CXCL-8: C-X-C Chemokine receptor type eight; CXCL-9: C-X-C Chemokine receptor type nine; CXCL-10: C-X-C Chemokine receptor type ten; CXCL-3: C-X-C Chemokine receptor type tree; D3: Day 3; D4: Day 4; D5: Day 5; D6: Day 6; D7: Day 7; D15: Day 15; D30: Day 30; FAPEMIG: Fundação de Amparo à Pesquisa do estado de Minas Gerais; FIOCRUZ: Fundação Oswaldo Cruz; IFN- γ: Interferon gamma; IL-8: Interleukin eight; IL-5: Interleukin five; IL-4: Interleukin four; IL-10: Interleukin ten; IL-2: Interleukin two; IL-10: Interleukin 10; IP-10: Interferon gamma-induced protein ten; IU: International unit; LATEV: Laboratório de Tecnologia Virológica de Bio-Manguinhos; Log_10_: Logaritmo ten; MCP-1: Monocyte chemotactic protein-1; MFI: Mean fluorescence intensity; MHC: Major histocompatibility complex; MIG: Monokine induced by gamma interferon; mIU/ml: Milli- international units per milliliter; MIP-1 β: Macrophage inflammatory protein 1 beta; n: Number; PCR: Polymerase chain reaction; PDTIS: Rede de Plataformas Tecnológicas do Programa de Desenvolvimento Tecnológico em Insumos para Saúde; PFU: Forming unit board; PNI: Programa Nacional de Imunização; PRNT: Plaque reduction neutralization test; PROEP: Programa de Expansão da Educação Profissional; PV: Primary vaccination; RNA: Ribonucleic acid; SVS: Secretariat of health surveillance; TNF- α: Tumor necrosis factors alpha; WHO: World health organization; YF: Yellow fever; YF NS5: Region five of yellow fever virus.

## Competing interests

The authors declare that they have no competing interests.

## Authors’ contributions

Thirteen authors (MLSM, RHGF, MSF, RG, AMYY, LFCA, SMBL, GRSS, DAH, RC, MLFL, AH and RMM) are employees at the 17DD-YF vaccine manufacturer (Bio-Manguinhos, Fundação Oswaldo Cruz), and five authors work in other units of Fundação Oswaldo Cruz (VPM, LABC, RMRN, ATC and OAMF). Bias from competing interest was prevented by: (1) collaboration of two general clinical physicians (RAVF and EPF) from Instituto de Biologia do Exército; Rio de Janeiro, Brazil, with experience in infectious diseases. RAVF and EPF contributed with critical overview of the study design, volunteers’ immunization and medical care, blood sample collection, and supervision of data interpretation. The Collaborative Group for the Study of Yellow Fever Vaccines was responsible for carrying out the study and (2) inclusion of two independent university professionals working as Undergraduate students (PAE and GVR) or Post-doctoral researchers (ACCA and JGCR) in the field of infectious diseases, responsible for blind sample handling and processing, data collection, and statistical analysis. All authors have read and approved the final manuscript.

## Pre-publication history

The pre-publication history for this paper can be accessed here:

http://www.biomedcentral.com/1471-2334/14/391/prepub

## Supplementary Material

Additional file 1: Figure S1 Persistence of Anti-YF neutralizing antibody titers one year after 17DD-YF primary vaccination with different doses (27,476 IU-current; 10,447 IU; 3,013 IU; 587 IU; 158 IU and 31 IU). PRNT assay was carried out 365 days (D365) after primary vaccination, as described in Methods. PRNT antibody titers (• = current dose and fades for subdoses) are expressed in log_10_ mIU/mL and 2.7 log_10_mIU/mL as the cut-off point to segregate seropositive from seronegative samples. Significant persistence of seropositivity (>95%) as compared to D30 is highlighted by gray rectangle. Click here for file
