# Supplementary figures and images for: Subdoses of 17DD yellow fever vaccine elicit equivalent virological/immunological kinetics timeline
Source: BMC Infect Dis. 2014 Jul 15;14:391. doi: 10.1186/1471-2334-14-391 (PMC4223624; doi:10.1186/1471-2334-14-391)

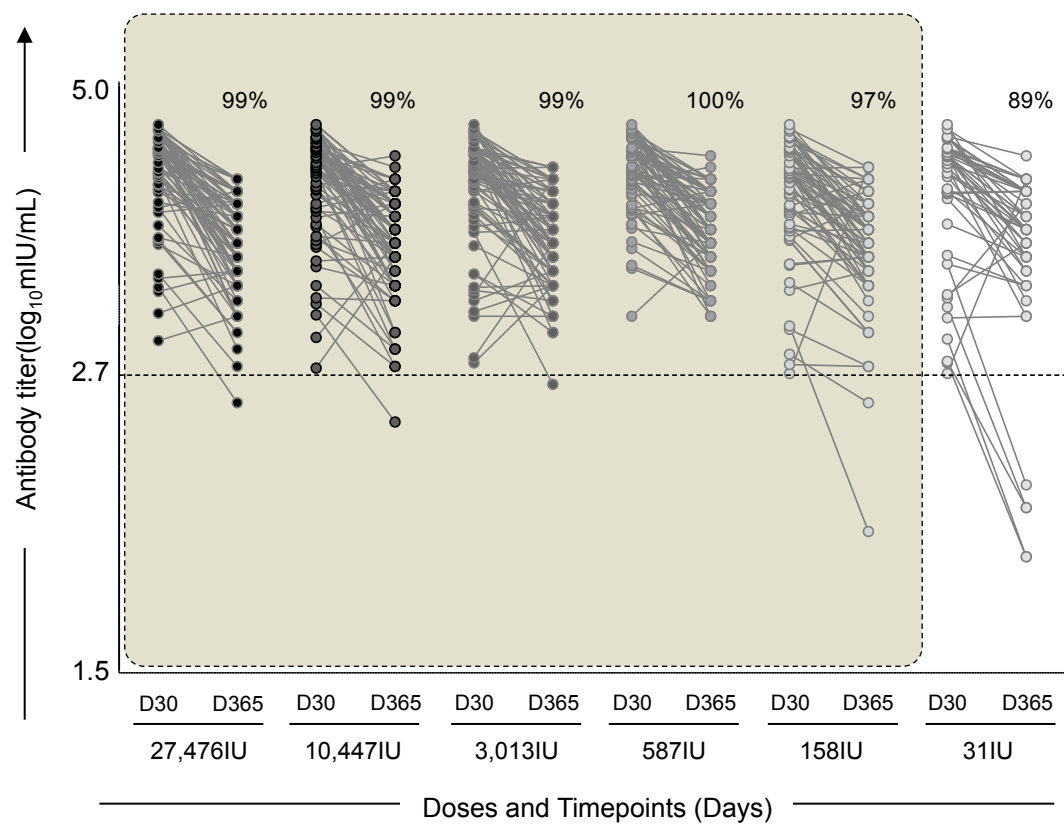

Supplement: Additional file 1: Figure S1 — Persistence of Anti-YF neutralizing antibody titers one year after 17DD-YF primary vaccination with different doses (27,476 IU-current; 10,447 IU; 3,013 IU; 587 IU; 158 IU and 31 IU). PRNT assay was carried out 365 days (D365) after primary vaccination, as described in Methods. PRNT antibody titers (• = current dose and fades for subdoses) are expressed in log10 mIU/mL and 2.7 log10mIU/mL as the cut-off point to segregate seropositive from seronegative samples. Significant persistence of seropositivity (>95%) as compared to D30 is highlighted by gray rectangle. [file 1471-2334-14-391-S1.pdf]
